# What It Means to Be Oneself: The Everyday Ideas of Authenticity among Primary School Children and Adolescents in Russia

**DOI:** 10.11621/pir.2021.0301

**Published:** 2021-09-15

**Authors:** Sofya K. Nartova-Bochaver, Roksana M. Bayramyan, Kirill S. Chulyukin, Victoria G. Yerofeyeva

**Affiliations:** a HSE University, Moscow, Russia

**Keywords:** authenticity, true self, adolescents, everyday representations, descriptors, content analysis

## Abstract

**Background:**

Personal authenticity is a person’s ability to be oneself and coherent in both his/her personality and the circumstances of his/her life (time, place, and life-calling). The sense of one’s true self plays an essential role in peoples’ psychological well-being and life goals. Currently, the theory of authenticity is included in existential psychology, the person-centered approach, and the psychology of the subject, but all of these approaches have some methodological limitations.

**Objective:**

The aim of the current study was to explore the everyday presentations of the true self among the primary school children and adolescents. It was expected that in adolescence, these representations are more differentiated and mature than at an earlier stage of life.

**Design:**

In the exploratory research, 330 respondents took part, including 163 primary school children (74 girls, 87 boys, ages 7 to 11; M = 9.4) and 167 adolescents (78 girls, 89 boys, ages 12 to 17; M = 14.3). A special interview consisting of 11 open and closed-ended questions was developed. The inductive method of content analysis was used.

**Results:**

Differences were found in the frequencies of the categories used by primary school children and adolescents. Older respondents described their true selves in more detail; their evaluations were more positive and often included their social life as an inseparable part of themselves, whereas descriptions by the younger children were more sparse, ambiguous, and individualistic.

**Conclusion:**

The results obtained can help identify the substantial stages of the genesis of the true self. To develop authenticity, these facts should be taken into consideration.

## Introduction

Authenticity, as the ability to be oneself or to follow one’s true self, is defined in our study as the coherence among a person’s life experiences (actions, cognitions, and emotions), his/her personality (temperament, values, and beliefs), and the circumstances of his/her life (time, place, and life-calling). The need to be authentic is a constant value in various forms in all cultures and generations. Authenticity lets a person be resilient, emotionally stable, and psychologically healthy; enables friendships; and leads to a meaningful moral life (Brunell et al., 2010; Chessick, 1996; Goldner & Berenshtein-Dagan, 2016; Harter, 2002; Impett, Sorsoli, Schooler, Henson, & Tolman, 2008; Mengers, 2014; Peets & Hodges, 2018; Rivera et al., 2019; Thomaes, Sedikides, van den Bos, Hutteman, & Reijntjes, 2017; Tsang, Hui, & Law, 2019; Yanchenko & Nartova-Bochaver, 2020; Zhang et al., 2019).

The opposite is also true: people who lack in personal authenticity are prone to addictions, depressions, and immoral attitudes toward the world (Anli, 2018; Gino, Kouchaki, & Galinsky, 2015; Vargová, Zibrínová, & Baník, 2019). Hence, people who want to become self-actualized set a goal to develop authenticity. The concept has not been studied comprehensively, and this phenomenon has never been in the mainstream of personality ref45research; it steadily retains a somewhat marginal and complex nature, but remains as a very attractive subject for researchers (Baumeister, 2019; Newman, 2019; Strohminger, Knobe, & Newman, 2017).

Moreover, authenticity is recognized as one of the most problematic, troubling, elusive, and vague concepts of personality psychology (Jones, 2010; Hicks, Schlegel, & Newman, 2019). We can offer some logical explanations for this.

First, the ontological status of authenticity in psychology is not obvious: it is considered a personality trait, a state, or a contextual characteristic (Kernis & Goldman, 2006; Lehman, O’Connor, & Carroll, 2019; Lenton, Slabu, & Sedikides, 2016; Robinson, Lopez, Ramos, & Nartova-Bochaver, 2013; Wood, Linley, Maltby, Baliousis, & Joseph, 2008; Sparby, Edelhäuser, & Weger, 2019).

Second, it can be investigated from both interpersonal and intrapersonal perspectives (Grégoire, Baron, Ménard, & Lachance, 2014).

Third, it is assumed that the content differs depending on the paradigm and culture in which it is studied (Ito & Kodama, 2007; Xia, Lv, & Xu, 2021). Moreover, some researchers are sure that authenticity cannot be identified by scientific concepts at all, because it does not meet the standards of objectivity and verifiability (Strohminger et al., 2017).

Nevertheless, despite this relative ambiguity of understanding, authenticity continues to be the subject of lay theories, folk representations, and everyday reality in the humanities branch of personality psychology and psychotherapy. Our work contributes to this line of research.

### Authenticity in different paradigms of personality psychology

Contemporary psychology emphasizes several manifestations of personal authenticity. According to Harter, it involves “owning one’s personal experiences, be they thoughts, emotions, needs, wants, preferences, or beliefs, processes captured by the injunction to ‘know oneself.’” (Harter, 2002, p. 382). Kernis & Goldman (2006), using the existential paradigm of personality research, suggest that authentic functioning should include four components: 1) self-understanding; 2) recognizing one’s ontological realities objectively; 3) behavioral actions; and 4) certain features of interpersonal relationships. In the humanistic person-centered conception by Rogers (1951), authenticity is the degree to which a person’s primary experience, symbolized awareness, and outward behavior and communication correspond to each other (Barrett-Lennard, 1998; Nartova-Bochaver, Reznichenko, & Maltby, 2020; Wood et al., 2008). Authenticity itself is expressed in the ability to live authentically without accepting external influence, and the absence of self-alienation. Sheldon, Ryan, Rawsthorne, & Ilardi (1997) consider authenticity a sign of a person’s self-organization (in contrast to cross-role variation as a mark of disorganization); this idea has been being developed nowadays (Ryan & Ryan, 2019).

Most of these definitions, except for the Kernis and Goldman (2006) theory, highlight, in full accordance with the western tradition, the internal consistency of the individual and the inter-correspondence of their desires, opinions, roles, decisions, and behavior. It is noteworthy that Rogers represented a very individualistic position in the personality psychology, and the influence of the surrounding world was clearly interpreted by him as a threat to authenticity (Strohminger et al., 2017; Sedikides, Lenton, Slabu, & Thomaes, 2019).

Since Russia is situated between East and West, it combines features of individualism and collectivism in its culture. Therefore, we expect that the range of everyday ideas about authenticity will include both points of view. People in the eastern collectivistic cultures are eager to establish relationships with people and mutual obligations (Kagitcibasi, 2013). As Xia et al. (2021) noted, people from collectivistic cultures are more likely to accept external influence and integrate it as a part of their sense of self, their true self.

According to Rubinstein’s (2012) subject-activity approach, people are inseparable from the world, including the social one. Hence, their experience of authenticity (the true self) might reflect the “correct” quality of relations with the world, like a sense of their relevance in time and space, compliance with life’s vocation, and acceptance of fate (Nartova-Bochaver, 2011). Supporters of the holistic eastern understanding of a person as part of the world define authenticity as harmony between oneself and one’s life course, including destiny (Leontiev & Shilmanskaya, 2019).

Moreover, manifestations of personal authenticity are even trickier: sometimes actions in accordance with one’s own personality are felt and considered authentic, and sometimes they are rather complementary to personality (for example, an introvert considers his/her extroverted actions as authentic ones) (Fleeson & Wilt, 2010). In other words, authenticity can be identified, experienced, and comprehended “from the opposite,” in non-typical or unexpected situations.

Summarizing their research on the true self, Strohminger et al. (2017) identified its four features as follows: 1) the true self emphasizes moral features; 2) it is valence-dependent and positive by default; 3) the true self is perspective-independent and does not vary depending on whom the person describes himself/herself to; and 4) it is cross-culturally stable. These features represent the true self as an archetype rather than as a cultural and age-sensitive phenomenon (whereas the rest of research considers authenticity just this way) and, therefore, it can hardly be studied within the framework of evidence-based positivist thinking.

### The development of authenticity in children of school ages: first approaches

As our quick introduction showed, the investigation of the true self has a great number of gaps and blanks, so researchers must deal with contradictions and confusions. The genesis of authenticity and the dynamics of its ideas in everyday life is one of the most gaping lacunae. According to Rogers’ person-centered approach, as a child, a person lives in harmony, feels authentic in accordance with his/her nature, relying on his/her organismic valuingand being free from outside influences (Rogers, 1951). As a child matures, the balance between trusting him or herself and other people’s assessments changes, and he/she loses the belief in his or her own feelings, and starts to trust others. School, the first social institution, restricts the child’s freedom by means of rules.

According to Erikson (1963), during the elementary school stage (ages 6–12), children face the choice ofindustry vs. inferiority.They compare themselves with their classmates, and are either proud of themselves or feel inferior. All these processes contribute to the development of their senses of self and the true self, which increases at the adolescence stage (ages 12–18), when children must resolve the question of identity vs. role confusion.

Adolescents struggle with questions regarding who they are, and what they want to do in their lives. Researchers consider adolescence as the most crucial period for the development of ideas about one’s true self (authenticity). Children of this age become especially sensitive to any manifestations of insincerity and learn to distinguish between the true and false self: “Although factors influencing authenticity begin in childhood, not until adolescence are individuals actively interested in, if not concerned about, whether their behavior reflects true-self or false-self behavior” (Harter, 2002, p. 384). Consequently, it’s at this life stage that people become aware of their true selves, and make an effort to achieve authenticity (Tsang et al., 2012; Uhlendorff, 2004). Authenticity benefits adolescents: it enhances their sense of well-being, covaries with satisfaction of psychological needs for relatedness and competence, and mediates the relationship between need satisfaction and well-being (Heppner, Kernis, Nezlek, Foster, Lakey, & Goldman, 2008; Thomaes et al., 2017).

One of the first studies of adolescents’ authenticity, conducted on the sample of respondents ages 12 to 18, showed that young adolescents felt most authentic only when disclosing single facts of their lives to others (sincerity), whereas older teens recognized and expressed their “true” nature not occasionally, but constantly, regardless of how other people see them. (Ullman, 1987).

At the same time, Harter, Waters, Whitesell, and Kastelic (1997) noted that adolescents manifested different personalities depending upon whether they were interacting with their mother, father, close friends, romantic partners, or peers; this fact represented their multiple selves. Thus, they felt cheerless with family, happy in a group of friends, shy with a partner, open in a group of close friends, hardworking at school, reliable at work, or naughty and less responsible with peers. Some adolescents are not confused or embarrassed when they behave in different ways (Weir & Jose, 2010). That is because one of the critical developmental tasks in adolescence is to create multiple selves, along with the true self (Harter, Bresnick, Bouchey, & Whitesell, 1997). Developing multiple selves does not imply the cultivation of the false self. While having multiple selves is considered a normal developmental process, a false self is alluded to as the result of self-alienation. A false self is “the extent to which one is acting in ways that do not reflect one’s true self as a person or the “real me.”” (Harter, Marold, Whitesell, & Cobbs, 1996, p. 360).

Self-alienation has been investigated more vigorously than authenticity, since negative phenomena are usually presented in everyday life more varied and can be understood more deeply (Baumeister, Bratslavsky, Finkenauer, & Vohs, 2001). So, Harter et al. (1996) identified three parental and peer support variables which predict adolescents’ false or true selves, namely: 1) level of support; 2) quality of support (unconditional or conditional); and 3) hope about future support. If adolescents lack these resources, they are more likely to feel self-alienated. A lower rate of self-esteem and a higher level of anxiety also contributed to the risk of false-self behavior and, as a result, the false self (Vargová et al., 2019). Rayce, Holstein, and Kreiner (2009) distinguished alienation of thoughts, actions, or personality in general, and proposed to calculate an index of alienation. O’Donnell, Schwab-Stone, and Ruchkin (2006) emphasized the role of alienation and self-estrangement in maladjustment due to being exposed to community violence.

As for the conditions for achieving authenticity, the main one is Rogers’s (1951) concept of unconditional positive regard. The rest of predictors stimulating authenticity are warm relationships with parents (Theran, 2009) and security within the family (Goldner & Berenshtein-Dagan, 2016); emotion management skills (Gross & John, 2003); satisfaction of needs, especially the need for autonomy (Thomaes et al., 2017); true-self behavior with parents and classmates; and knowledge of one’s true self (Goldner, & Berenshtein-Dagan, 2016). Some researchers consider the school environment a threat to the authenticity of adolescents whereas homeschooling could protect them (Brown, Higgins, & Paulsen, 2003; Sarajlic, 2019).

In summary, over the course of children’s school life, the true self (personal authenticity) turns into a value which can be gained through effort. Although data about the adolescents’ sense of true self is lacking, we assumed that these representations are becoming more nuanced and differentiated, compared with primary school children. Adolescents also include the true self protectors, like figures of admired others, in their experience of the true self, along with threats of its violation (like school obligations), and its consequences for later life.

Our research was exploratory. We did not put forward explicit hypotheses because of the data deficit. Instead, we raised several research questions:

1. How do adolescents cognitively understand the meaning of “to be oneself ”?
2. What role do other people play in adolescents’ feelings of the “true self ”?
3. What is more tangible for the true self: self-manifestation or self-alienation?
4. How do they emotionally evaluate themselves and their lives?

Our empirical research was devoted to obtaining answers to these questions.

## Method

We followed a bottom-up approach typical for an exploratory assessment (Kovács, 2019; Ullman, 1987) because of the absence of a generally accepted definition of authenticity. To study the descriptors of authenticity in two groups (*i.e.*, primary school children and older adolescents), a special structural interview was developed. Kernis and Goldman (2006) offered four categories: awareness; biased self-esteem; behavior; and orientation in relationships. Wood et al. (2008) singled out three scales: authentic living, accepting external influence, and self-alienation.

After we analyzed these parameters and discussed the definitions and wordings, we created six iterations. Open questions were developed for five units: 1) authentic living; 2) accepting external influence; 3) self-alienation; 4) authentic behavior; and 5) balance between social and individual. Every subscale included two or three questions; the full interview consisted of 11 questions, five of which were open-ended, five close-ended, and one consisting of two parts, one open and one closed.

Two versions of the interview were prepared, one for primary school children and one for adolescents, both with the same meaning of the questions. A written form of the interview was chosen as a means of minimizing the influence of others, letting the respondents think deeply about themselves, and being more convenient for further analysis. Writing practices are a useful tool for self-reflection (Murray, 2002; Tartakovsky, 2015); in addition, our interview seemed to have a small psychotherapeutic effect.

To process the data, we chose qualitative content analysis, namely, the inductive method that implied finding codes and forming categories from the data without a pre-established theory (Gondim & Bendassolli, 2014; Mayring, 2000). As the codes were extracted from the data (Hsieh & Shannon, 2005; Busygina, 2013), conventional qualitative analysis was most relevant.

Two people coded the data independently to assure the analysis’s validity and reliablility (Bengtsson, 2016; Erlingsson & Brysiewicz, 2017; Yardley, 2000). Similar codes were enlarged and spliced (Marks & Yardley, 2004). The categories emerged based on the code tables (White & Marsh, 2006). One independent expert performed the same task as the coder. To eliminate any ambivalence, the coders discussed the results (Yardley, 2000). After that, the coding system was checked, and the final categories were approved by the research group.

The condensation of meaning technique was used to go from lower levels of abstraction to higher ones (Erlingsson & Brysiewicz, 2017; Kvale, 2003). A divided coding system, when several codes were found in one respondent’s answer, was chosen (Marks & Yardley, 2004). Therefore, the number of codes in categories of open-ended questions differs from the overall quantity of respondents.

We compar ed groups for the following parameters: 1) *cognitive appraisal of the true self;* 2) *relationships with others (dichotomy “I vs. others”);* 3) *sense of the true self;* and 4) *emotional perception of one’s true self and life* (see Appendix). We used the Chi-squared test to evaluate the differences between the categories. The unique categories were excluded from the analysis if they were mentioned by only one group (Chi-squared test for nominal (categorical) data, 2021). We arranged the five answers in one group to form the independent category. The codes that did not align with any category were grouped into the category *Different.*

### Participants

330 respondents took part in our research: 163 primary school children (74 girls, 87 boys, and 2 who did not identify their gender; ages 7 to 11; M = 9.4) and 167 adolescents (78 girls and 89 boys; ages 12 to 17; M =14.3).

### Procedure

The study was conducted at the Russian-Tatar secondary schools in the multinational large city of Kazan. The so-called front-individual survey was used, taking into account the age characteristics of primary school children and adolescents. We conducted the survey in person, in class. We set the task and time limits at 15-20 minutes; the purpose of the interview was presented as evaluating the children’s understandings of themselves and their lives. The children answered using the pencil/paper technique; they did not reveal their names. The content of the interview aroused the students’ interest, provoking them to ask questions aimed at understanding their behavior, typical life situations, and share their assumptions and conclusions.

## Results

As the age groups were similar in size, we show the initial numbers of categories in the tables. All the adolescents’ answers were much more detailed, providing 8,509 words in total, or 51 words for each respondent on average. For primary school children, the data set included 5,402 words, or 33 words for each respondent on average.

### Cognitive appraisal of the true self

Eleven categories were singled out in the primary school children group, and 10 in the adolescent one (*Table 1*). The frequencies of categories between groups differed significantly (Chi-squared = 72.47; df = 8; p< 0.01). A large number of the primary school children had problems with defining what it meant “to be oneself,” so the categories *No answer* and *Different* were most frequently chosen.

**Table 1.** Cognitive appraisal of the true self

The categories	Primary school children / Adolescents
No answer	35 / 18
Different	34 / 15
Independent behavior	28 / 30
Independent thoughts	3 / 33
Self-identity/Not to create the false self	11 / 49
Feelings and attitude to oneself	10 / 30
To be individual	10 / 3
Solitude	6 / 3
*Authenticity as a value*	14 / 0
*To be a human*	5 / 0
*Near other people*	0 / 11

*Note. Italics indicates categories that occurred only in one group (excluded from the Chi-squared test)*

*Independent behavior* turned out to be practically equal in two groups, whereas among adolescents, *Independent thoughts* were broadly represented. Most wanted to save their *Self-identities* and *Not create the false selves*, although primary school children did not identify these categories as the most essential aspects of being themselves. Primary school children denoted *To be individual* and *Solitude* more often than adolescents. *To be with other people* was a category that made its appearance with the adolescents group for the first time. Younger children preferred unique categories, like *Authenticity as a value* and *To be a human* contrary to adolescents’ *Near other people.*

### Relationships with others (Dichotomy “I vs. others”)

When a person tries to be authentic, he or she has to resolve the conflict between two inclinations: the inner vs. outer and him/herself vs. others. Then he/she must find the optimal balance ( *Table 2*). Primary school children supposed that other people helped them understand themselves, but adolescents were convinced that others played only a moderate role in helping them discover themselves (Chi-squared = 32.00; df = 5; p< 0.01). Only adolescents identified the *Self-orientation* category.

**Table 2.** Relationships with others (Dichotomy “I vs. others”)

The categories	Primary school children / Adolescents
1. Do other people help or interfere with understanding oneself?**	
Help / Rather help	71 / 48
Interfere / Rather interfere	23 / 22
Help and interfere	12 / 13
Do not interfere (Indifferent attitude)	15 / 9
No answer	24 / 8
Different	18 / 52
*Self-Orientation*	0 / 15
2. The commitment to one’s own point of view**	
Follow own opinion	93 / 103
Rather follow own opinion	2 / 17
Rather not follow own opinion	3 / 14
Do not follow	23 / 10
Different	14 / 10
No answer	28 / 13
3. Listen to oneself or others**	
Listen to oneself	63 / 83
Listen to oneself or others	12 / 23
Listen to others	27 / 10
Different	27 / 27
No answer	34 / 24
4. Feeling Authentic when alone or with others**	
The same feelings whether alone or with others	61 / 80
Alone	14 / 11
With others	7 / 8
No (without differentiation)	19 / 26
Different	26 / 5
No answer	36 / 22
*Ambivalent feelings*	0 / 15

Note. ** Differences are significant at p< 0.01 *Italics indicates categories that occurred only in one group (excluded from the Chi-squared test)*

Most respondents in both groups did not change their minds when their opinions differed from the others’ views (Chi-squared = 30.73; df = 5; p< 0.01). They listened to their own voices more often than other people’s (Chi-squared = 15.64; df = 4; p< 0.01). Moreover, older respondents were becoming more independent; they trusted themselves more than others.

Most respondents’ feelings did not differ depending upon whether they were alone or with other people; however, in adolescents, such answers were much more frequent (Chi-squared = 21.34; df = 5; p< 0.01). Adolescents emphasized their ambivalent feelings toward others, while primary school children did not.

*The Sense of the true Self:* This theme includes two parts, self-manifestation and self-alienation (*Tables 3a* and *3b*). Most respondents from both groups did not know what type of their behavior reflected their true selves and what type of behavior did not. Adolescents emphasized twice as often as primary school children that they always showed their true selves (Chi-squared = 33.81; df = 5; p< 0.01). They needed more contact with others and their peers; when being alone, their sense of true self decreased. The category *Being a subject of the action* was identified only in that group. Adolescents identified *Games and other activities* as more inherent to their true selves than the younger group. *Independent choice of activity* and *Listening to one’s own voice* is the sources of authenticity which appear at primary school age.

**Table 3a:** The sense of the true self: self-manifestation

The categories	Primary school children / Adolescents
1. Situations when a person feels the True Self**	
No answer	45 / 42
Always	19 / 38
Different	49 / 25
*Independent choice of activity*	14 / 0
At home	13 / 18
Being alone	12 / 5
Contact with others and their confession	11 / 40
*Walking*	6 / 0
*Lack of anxiety*	5 / 0
*Listening to one’s own voice*	5 / 0
*Being a subject of the action*	0 / 9
*Games and different activities*	0 / 7
2. Behavior that reflects respondents’ personality**	
No answer	70 / 53
Different	69 / 41
Good deeds	14 / 17
Always	8 / 23
At school	5 / 5
*Being a subject of the action*	0 / 9
*At difficult life situations*	0 / 8
*In communication*	0 / 7
*Doing the activity that a person likes*	0 / 6
*In quarrels*	0 / 6

Note. ** Differences are significant at p< 0.01 *Italics indicates categories that occurred only in one group (excluded from the Chi-squared test)*

**Table 3b:** The sense of the true self: self-alienation

The categories	Primary school children / Adolescents
3. Situations when a person cannot understand themselves**	
No answer	41 / 25
Do not lose myself	26 / 78
Different	46 / 15
*When a person has unpleasant feelings*	16 / 0
When vulnerable to others	11 / 30
At school	9 / 3
When being aggressive	6 / 12
*Among other people*	0 / 7
*In the street*	6 / 12
*In bad situations*	5 / 0
4. Means of self-recovery**	
No answer	69 / 59
Different	41 / 33
When a person relaxes	20 / 13
*Without awareness*	9 / 0
Inner monologue	9 / 7
Among other people	7 / 14
Doing the activity one likes	6 / 6
Being alone	4 / 7
No self-alienation	5 / 34
*Games*	5 / 0

Note.** Differences are significant at p< 0.01 *Italics indicates categories that occurred only in one group (excluded from the Chi-squared test)*

Although most respondents did not understand what actions best reflected their personalities, adolescents had a much broader repertoire of actions and situations that corresponded to their true selves, such as *Personal responsibility, Difficult life situations, Communication, Favorite activity*, and *Quarrels* (Chi-squared =14.75; df = 4; p< 0.01).

As for self-alienation (the opposite of being authentic), adolescents, compared with primary school children, seldom felt lost (Chi-squared = 58.10; df = 5; p< 0.01). Emotions were one of the key points for defining one’s true self. Adolescents, unlike primary school children, felt annoyed or upset when they were aggressive toward others, or were among other people. Whereas school children met their inner selves and learned to accept their feelings that might be unpleasant or negative, adolescents rather focused on judging themselves. In addition, some primary school children said that school was an unsafe place for their psychological well-being.

Identifying resources for the recovery of authenticity was a very difficult task for both groups; nevertheless, their answers differed (Chi-squared = 27.22; df = 7; p< 0.01). *Relaxation*, *Being among other people,* and *Doing the activity that one likes* were more valued among primary school children; *Games* were identified as a separate category only in this group. For adolescents, the role of other people was ambivalent: they preferred to share the time with others, and chose to stay alone twice more often than the primary school children did. In addition, they often reported the absence of self-alienation.

### Emotional perception of one’s true self and life

Finally, we contemplated that authenticity might appear when a person becomes immersed in him or herself, which practice helps people meet their true selves (*Table 4*). As expected, this category significantly differentiated primary school children and adolescents; with a high presence of undefined descriptors in both groups, adolescents accepted and liked themselves more than primary school children did (Chi-squared = 9.39; df = 3; p< 0.05).

**Table 4.** Emotional perception of one’s true self and life

The categories	Primary school children / Adolescents
1. Emotional appraisal of one’s inner world*	
Like	106 / 131
Not very	19 / 7
Do not like	15 / 12
No answer	23 / 17
**2. Emotional perception of one’s life**	
Like	112 / 115
Not very	14 / 19
Do not like	9 / 7
Different	5 / 3
No answer	23 / 23
**3. Aspects of life which respondents liked**	
*Like, because life is good*	14 / 0
Relationships with family and friends	9 / 21
Different	19 / 30
Everything	12 / 23

Note.* Differences are significant at p< 0.05 *Italics indicates categories that occurred only in one group (excluded from the chi-squared test)*

Furthermore, most participants were satisfied with their lives, and there were no differences between age groups on this parameter (Chi-squared = 2.02 < 9.49 at df = 4 and p = 0.05). Finally, most respondents ignored the question about the most liked aspects of their life (Chi-squared = 0.64 < 7.82 at df = 3 and p< 0.05).

## Discussion

Our analysis of ideas about the true self has shown that it is not an archetype but a core component of the empirical self, which is sometimes moral, sometimes immoral, manifests itself in typical and special situations, and is able to develop with growing up.

The results we obtained answered all the research questions raised in the introduction. As expected, everyday ideas of the person’s sense of the true self are becoming better developed in adolescence compared to primary school age. Four categories identified during the content analysis of respondents’ comments (*cognitive appraisal of the true self; relationships with others; the sense of the true self;* and *emotional perception of one’s true self and life*) really differentiated respondents from the two age groups; these differences were both quantitative and qualitative. The adolescents’ answers were more differentiated and nuanced. Primary school children marked authenticity as a value and a side of their personality, whereas adolescents associated authenticity with separation from others, predominantly adults, and inclusion of significant others in their true selves.

As for identifying the best situations for manifestation of one’s true self and the signs of it, older children mentioned being among other people and independent thinking, whereas younger ones brought up solitude and independent behavior only. It may be that the feelings and attitudes toward oneself are becoming more differentiated as people become older, and their cognitive complexity rises (Kelly, 1955). In addition, becoming an authentic personality is closely connected with entering the worlds of others (Rubinstein, 2012).

The analysis of the category *Relationships with others (Dichotomy “I vs. others”)* showed that the adolescents observed the changes in their relationships with others: they trusted themselves, integrated various feelings toward others, and tried to find the balance between “I” and “they.” All these steps are essential for dividing the inner world from the outer. Adolescents start listening to their own voices more often than other people’s. This is in line with Harter’s et al. (1998) results, which demonstrated that having one’s own voice is a crucial indicator of the true self in adolescence, and is bound to adolescents’ independent thinking as a manifestation of their personal authenticity.

As for the category *Sense of the true self*, most respondents in both groups were not aware of which were the situations and behaviors when their true selves appeared. Regarding self-alienation, they demonstrated important differences: whereas primary school children lost a sense of themselves in situations where they were victims (in the school or a street; generally, in “bad” situations), adolescents felt self-alienated when they themselves were agents of aggression Hence, they realized that they could offend others and distanced themselves most when they were vulnerable to others. These facts are easily interpreted, considering that adolescents appreciate social connections much more than primary school children do.

Finally, analysis of the category *emotional perception of one’s true self and life* showed that both children and adolescents failed in being able to interpret their feelings; the observer self had not yet been formed. However, they were engaged in their private lives, and generally, most respondents were satisfied with both their true selves and lives. Some difficulties in describing their experience existed because they were still developing cognitively, emotionally, and personally. The fact that most respondents ignored the question about the most liked aspects of their true selves demonstrates a lack of reflection and perspective-taking, which might be overcome at the next stage of development.

Now, we can answer the research questions raised in our study.

1. How do adolescents and primary school children cognitively understand the meaning of “to be oneself ”? Younger respondents associate the true self with independent actions, while older ones try to show their real selves to others.
2. What role do other people play in adolescents’ feelings of the “true self ”? The opposition between the individual self and other people, proclaimed by Rogers, has not been shown: the respondents in both groups listened to their inner voices and built strong and enriching relationships with others.
3. What is more tangible for the true self, self-manifestation or self-alienation? As for the sense of the true self, it is more easily felt and understood in self-manifestation than in self-alienation. We concluded that adolescents and primary school children had never thought about losing their sense of self.
4. How do they emotionally evaluate themselves and their lives? Most respondents appreciated life and themselves, and this positive feeling gives them an opportunity to accept themselves and becoming authentic.

To sum up, the sense of a true self is still far from being mature among both primary school children and adolescents. Their selves are not integrated, but authenticity is discovered in daily representations and experience. Moreover, contrary to Rogers’ (1951) belief that people are authentic only at an early age, and that they lose authenticity because others influence them, we have discovered that the sense of authenticity is enhanced by adolescence, and the social world makes a positive contribution to its maturation.

## Conclusion

The analysis of the descriptors among primary school children and adolescents allowed us to identify the main tendencies of personal authenticity development during these age periods. First, whereas the primary school children only recognized what it meant to be oneself, adolescents showed they already understood the concept when they chose to hide themselves. Secondly, associations with the true self stimulated by the interview questions were more positive in the older respondents. Finally, whereas in younger children the true self was connected with individualistic tendencies like looking for solitude, and a negative attitude toward the school, adolescents’ experiences of both authenticity and self-alienation were tightly bound to the social world, which is an inseparable part of their true selves.

The results obtained can be the basis for further research, perhaps with emerging adults.

## Appendix

### Cognitive appraisal of the true self

**Primary school children:**

“To be oneself is not to be like others.”

“Do what you want and spend the time you want.”

**Adolescents:**

“To be who I want to be, despite the opinions of other people.”

“It means not to betray your goals, beliefs, and principles, to defend them without imposing them, and to open up to people from the side that you consider necessary.”

### Relationships with others (Dichotomy “I vs. others”)

**Primary school children:**

“Best friends accept how I am me, and opponents look for my shortcoming.”

“I’d rather listen to myself, not others.”

**Adolescents:**

“My friends listen to my whining and all my problems. They accept me for who I am.”

“When I am alone, I feel sad, lonely, and when with other people, I feel important.”

### The Sense of the true self

#### Self-manifestation

**Primary school children:**

“When I do something good, kind.”

“When other people hear me, understand, do not ignore, do everything for me.”

**Adolescents:**

“I am myself everywhere: with friends, parents, in an unfamiliar society. I am comfortable with showing myself to other people.”

“In situations where I am worried or sad. I become like who I am inside myself.”

#### Self-alienation

**Primary school children:**

“When other people shout at me.”

“When I am scared.”

**Adolescents:**

“If I suddenly do something terrible, I will hate myself then.”

“I need to think and analyze the whole situation without the influence of anyone and motivate and love myself. But if you go wrong somewhere, you need to fix the situation and move on.”

### Emotional perception of one’s true self and life

**Primary school children:**

“I like myself.”

“I am neutral toward myself.”

**Adolescents:**

“I like my inner world.”

“I really like my fantasy; I plunge into a world in which I become happy, without any problems.”
